# Gallstone Hepatitis Caused by Transient Common Bile Duct Obstruction in a Middle-Aged Woman

**DOI:** 10.7759/cureus.30192

**Published:** 2022-10-11

**Authors:** Kotaro Murakami, Yudai Tanaka, Tsuyoshi Mishiro, Chiaki Sano, Ryuichi Ohta

**Affiliations:** 1 Family Medicine, Postgraduate Clinical Training Center, Shimane University Hospital, Izumo, JPN; 2 Community Care, Unnan City Hospital, Unnan, JPN; 3 Internal Medicine, Unnan City Hospital, Unnan, JPN; 4 Community Medicine Management, Shimane University Faculty of Medicine, Izumo, JPN

**Keywords:** japan, help-seeking behavior, liver enzymes, gallstone hepatitis, rural hospital, general medicine

## Abstract

The interpretation of hepatocholangial laboratory test results is challenging. Possible liver biochemical tests include the evaluation of alanine aminotransferase, aspartate aminotransferase, alkaline phosphatase, gamma-glutamyl transferase, 5'-nucleotidase, lactate dehydrogenase, bilirubin, and albumin levels, as well as prothrombin time and international normalized ratio. When liver enzyme levels are elevated, R-values are generally used for diagnosis. A 62-year-old woman presented to our hospital with the chief complaint of abdominal pain and was consequently diagnosed with gallstone hepatitis based on her blood test results. Generally, gallstone hepatitis manifests as elevated liver enzyme levels showing a hepatocellular pattern, while common bile duct obstructions show a cholestatic pattern. Since gallstone hepatitis is indistinguishable from viral and ischemic hepatitis in the early stages of onset, it is vital to monitor changes in symptoms, biochemical tests, and imaging results over time to diagnose this disease.

## Introduction

The interpretation of hepatocholangial laboratory test results is challenging. Liver biochemical tests usually include the assessment of alanine aminotransferase (ALT), aspartate aminotransferase (AST), alkaline phosphatase (ALP), gamma-glutamyl transferase (GGT), 5'-nucleotidase, lactate dehydrogenase (LDH), bilirubin, albumin (ALB) levels, and prothrombin time (PT)/international normalized ratio (INR) [1]. R-values represent a differential diagnostic approach for elevated liver enzyme levels. These values are calculated as follows: serum ALT level/upper limit of normal (ULN) divided by serum ALP level/ULN. An R-value ≥5 indicates a hepatocellular pattern, ≤2 indicates a cholestatic pattern, and 2-5 indicates a mixed pattern [1,2]. Viral hepatitis, ischemic liver disease, and toxic liver disease are the most common diseases that cause quickly elevated liver enzyme levels and exhibit a hepatocellular pattern [3]. In viral hepatitis, aminotransferase levels generally peak before the appearance of jaundice and subsequently decline slowly; consequently, the serum bilirubin level rises, and the LDH level only slightly increases in 50% of patients. In ischemic and toxic liver injuries, AST levels peak before ALT levels. The serum bilirubin level is generally less than 34 μmol/L, while the LDH level is very high [3].

Few hepatocellular diseases have transient courses [4]. These include gallstone hepatitis and a transient elevation of liver enzyme levels caused by gallstone obstruction of the biliary tract. When a common bile duct stone obstructs the biliary tract, liver enzyme levels are elevated in a cholestatic manner. However, acute obstruction without cholangitis may cause symptoms of hepatitis. In this study, we present the case of a patient with gallstone hepatitis whose hepatobiliary enzymes were monitored over time.

## Case presentation

A 62-year-old woman was admitted to our hospital with the chief complaint of abdominal pain and a prior history of hospitalization for ischemic enteritis for 4-10 days. Since being discharged from the hospital, she had been feeling well. She had abdominal distension on the afternoon of the day before the admission. After eating dinner, abdominal pain and nausea occurred simultaneously. She vomited every 30 min for one hour. She had cold sweats at midnight on the day of her admission. She visited our hospital with persistent abdominal pain. History-taking revealed that she drank alcohol only occasionally and did not eat oysters, deer, or wild boar, and did not consume any supplements, traditional Chinese medicines, or over-the-counter medicines. Her medical history included hypertension and hyperlipidemia. Prescribed medications included magnesium oxide (1320 mg), azilsartan (20 mg), amlodipine besylate (2.5 mg), rosuvastatin calcium (2.5 mg), and betamethasone ointment.

Her vital signs were as follows: body temperature, 36.4°C; pulse rate, 68 beats/min; blood pressure, 120/82 mmHg; and SpO_2_, 97% (room air). The abdomen was flat and soft, with tenderness in the epigastrium but no signs of peritoneal irritation. The blood test results are shown in Table 1. The patient was negative for viral acute hepatitis markers, with the hepatitis B (HB) antigen level of 0.00 IU/mL, hepatitis C virus (HCV) antibody level of 0.10 sample/cut-off (S/CO), and immunoglobulin M (IgM)-hepatitis A (HA) antibody level of <0.40 S/CO. At the time of admission 10 days prior, blood tests revealed no liver enzyme level elevation, which indicated an acute-onset elevation of liver enzyme levels (Table 1).

**Table 1:** Laboratory data in the clinical course Amy, pancreatic amylase; AST, aspartate aminotransferase; ALT, alanine aminotransferase; ALP, alkaline phosphatase; CK, creatinine kinase; CRP, C-reactive protein; GGT, gamma-glutamyl transferase (transpeptidase); LDH, lactate dehydrogenase; PT, prothrombin time; INR, international normalized ratio; aPTT, activated partial thromboplastin time

Day	-10	1	2	3	15
Albumin level (g/dL)		4.1			
Total bilirubin level (mg/dL)	0.4	1	1.1	0.6	0.5
AST level (IU/L)	27	867	462	141	24
ALT level (IU/L)	27	462	722	454	41
ALP level (U/L)	88	141	208	182	101
GGT level (IU/L)	26	165	272	238	116
LDH level (U/L)	230	827	412	197	165
Amy level (IU/L)		77			
CK level (U/L)		55			
CRP level (mg/dL)		0.16			
PT (%)		134.1			
PT/INR		0.86			
aPTT (s)		22.1			

**Figure 1:**
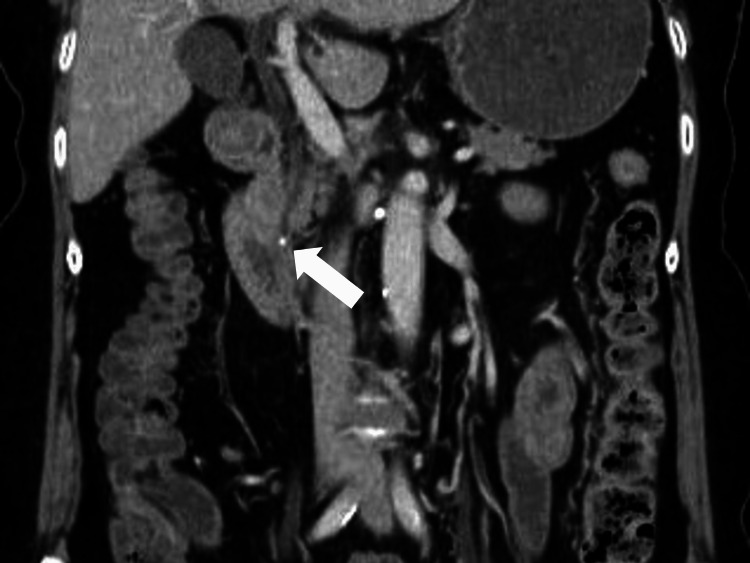
Abdominal computed tomography showing a stone in the lower bile duct (white arrow)

Contrast-enhanced computed tomography (CT) revealed no intrahepatic space-occupying lesions, and the margins of the liver were sharp and lacking in enlargement. The intrahepatic and extrahepatic bile ducts were not dilated, and two stones were found in the lower bile duct (Figure 1).

**Figure 2:**
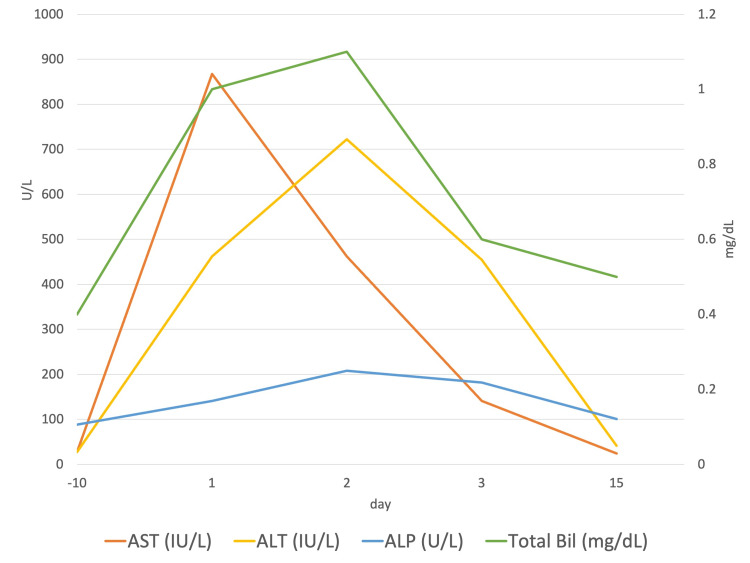
Time course of the laboratory data AST, aspartate aminotransferase; ALT, alanine aminotransferase; ALP, alkaline phosphatase; Bil, bilirubin

The patient was diagnosed with acute hepatitis due to elevated liver enzyme levels caused by the hepatocellular damage, with an R-value of 8.4, and was treated with bedrest, extracellular fluid infusion, and fasting. Her abdominal pain and soft stools were alleviated after admission. Blood tests showed reductions in liver enzyme levels (Figure 2).

CT performed on Day 3 showed that the common bile duct stones had passed spontaneously. The cause of acute hepatitis was unclear; nevertheless, the patient was discharged on Day 4, with full recovery of her symptoms. On Day 15, her liver enzyme levels improved (Table 1). The antinuclear antibody (ANA) nucleolar pattern increased 40-folds, whereas the anti-RNA polymerase was negative. The follow-up was completed without relapse until Day 22.

## Discussion

In the present case, the transient elevation of hepatobiliary enzyme levels was observed with acute-onset abdominal pain and nausea. On Day 1 of the onset, an AST level-dominant elevation was observed; however, on Day 2, the elevation was ALT level-dominant. The AST and ALT levels decreased on Day 2, while the ALP, GGT, and LDH levels increased. The common bile duct stones on Day 1 disappeared by Day 3, and the disease course was considered due to gallstone hepatitis.

In gallstone hepatitis, a gallstone obstructs the biliary tract, resulting in a transient elevation of liver enzyme levels in a hepatocellular pattern [4]. The disease presents at a relatively young age with acute severe abdominal pain, which often resolves within 48 h. Acute bile duct obstruction results in increased intraductal and biliary hydrostatic pressure. Aminotransferase levels are elevated because of bile acids' increased hepatocyte permeability and hepatocellular toxicity [5]. The bile duct was not dilated, and we could not determine whether bile duct obstruction occurred at the initial presentation. However, there is a difference in the incidence between young and elderly patients. The bile duct is thought to be more dilated in elderly patients than in young patients; thus, intraductal bile duct pressure may be less likely to increase. However, the risk of missing this disease must be considered because it also occurs in elderly individuals who may have poor complaints of pain.

Changes in biochemical test results due to biliary obstruction are generally marked by elevated ALP, GGT, and bilirubin levels. Generally speaking, within 24 hours after obstruction, AST and ALT levels are markedly elevated [6]. In toxic and ischemic hepatitis, AST levels are more elevated than ALT levels, which is believed to be due to a disturbance in zone 3 of the hepatic lobule. The half-life of AST is 17 hours, whereas that of ALT is 47 hours; this trend is reversed during the recovery period [7]. The course of the current case was similar, and it was difficult to distinguish gallstone hepatitis from acute hepatitis using biochemical tests alone. Furthermore, ALP levels have been reported to increase later than AST and ALT levels, while GGT levels after peak decline more slowly than AST and ALT levels [8]. In the present case, ALP and GGT levels increased later than AST and ALT levels. In most reported cases of gallstone hepatitis, liver enzyme levels decreased after conservative or invasive stone excision [5]. In this case, after liver enzyme levels had decreased, imaging studies showed gallstone excretion. Over time, the evaluation of biochemical test results may contribute to diagnosis when liver enzyme levels are elevated in a hepatocellular pattern.

In older patients with elevated liver enzyme levels, which are liver-damaging type, and acute hepatitis but no remarkable findings, follow-up physical examination and biochemical and imaging studies may lead to the suspicion and diagnosis of gallstone hepatitis [9,10]. Owing to the high incidence of elevated hepatobiliary enzymes and the absence of bile duct dilatation, various causes must be considered [11]. Clinical findings should be monitored over time to detect exacerbation of the condition or other symptoms, especially among older patients [12]. As invasive procedures are harmful and the benefit does not outweigh the risk of transient symptoms, magnetic resonance cholangiopancreatography (MRCP), endoscopic retrograde cholangiopancreatography (ERCP), and liver biopsy were not performed in the current case, which may indicate a weak basis for the diagnosis. Only contrast-enhanced CT was used to confirm the presence of gallstones, and only non-contrast-enhanced CT was used to confirm stone drainage. Invasive interventions can cause various complications, leading to morbidity and mortality; as such, we believe that MRCP, which is less invasive, is appropriate for diagnosing gallstone hepatitis.

## Conclusions

Gallstone hepatitis presents as acute severe abdominal pain at a relatively young age and shows a hepatocellular pattern with elevated liver enzyme levels. In this case, the diagnosis of gallstone hepatitis was based on changes in the symptoms, biochemical test results, and imaging results over time. As gallstones obstruct the biliary tract, GGT and ALP levels are also elevated, which differs from the manifestations of common acute hepatitis. Their elevation may be delayed compared to the fluctuations in AST and ALT levels. To diagnose gallstone hepatitis, it is important to monitor changes in symptoms, biochemical test results, and imaging results over time.
